# Intelligent Visual Prioritization for Retinal Prostheses via Context-Aware Object Ranking and Depth-Aware Phosphene Generation

**DOI:** 10.3390/biomimetics11090649

**Published:** 2026-09-09

**Authors:** Xinwei Li, Irshad Khalil, Faisal Rahman, Muhammad Nawaz Khan

**Affiliations:** 1 Department of Mathematics and Information Engineering, Liaocheng University Dongchang College, Liaocheng 252000, China; 2100061@lcudcc.edu.cn; 2 Department of Smart Security, Gachon University, Seongnam-si 13120, Republic of Korea; irshadkhalil@gachon.ac.kr; 3 Department of Information Technology Management, Southwest Baptist University, Bolivar, MO 65613, USA; faisal.rahman@sbuniv.edu

**Keywords:** retinal prosthesis, prosthetic vision, phosphene image generation, important-object selection, object ranking, context-aware learning, depth estimation, user preference modeling, Canny edge detection, simulated prosthetic vision, COCO dataset

## Abstract

Images from high-resolution cameras are mapped onto a sparse pattern of low spatial resolution and intensity in the retina, which limits visual perception in retinal prosthetic vision. When the entire scene is converted into phosphenes, it may allow unnecessary background information to be retained and may cause visual clutter, which may make it hard for prosthetic vision users to interpret the scene. In order to tackle this issue, this paper presents a context-, depth-, and user-preference-aware method for selecting the objects of interest in the generation of phosphene images. The proposed method does not show all the objects equally but learns to sort the objects according to their relevance to prosthetic vision. Manual annotation of a subset of COCO images was conducted where the most salient object was selected based on environment type, scene type, user mode, safety, navigation relevance, task importance, and distance. All of the candidate objects are described by full-scene visual features, object-crop features, handcrafted priority features, context embeddings, and monocular depth features. To predict object-level importance scores and identify the Top-1 and Top-4 important objects in unseen scenes, a hybrid deep learning model combining twin ResNet-18 backbones for scene and object feature extraction with embedding-based context encoding was trained. Priority maps and phosphene images were then created using the selected object masks and were depth-weighted. Two types of phosphene representations were also produced: Canny-edge-based and direct full images. The proposed framework is designed to suppress irrelevant background areas and improve important and closer objects in order to obtain a simplified and informative prosthetic-vision representation of the scene. The experimental evaluation, including Top-1 accuracy, Top-3 accuracy, mean reciprocal rank (MRR), and visual comparison, demonstrates the effectiveness of the proposed framework, achieving a Top-1 accuracy of 90.12%, a Top-3 accuracy of 97.45%, and an MRR of 0.9368. Furthermore, the proposed Canny-priority phosphene representation achieved an average human-participant recognition accuracy of approximately 86%. The proposed method offers a user-adaptive strategy for selecting and visualizing the information of a scene under the severe constraint of the bandwidth of retinal prosthetic vision.

## 1. Introduction

Blindness is a significant disability worldwide, affecting all aspects of life [1,2]. Visually impaired users were found to experience the greatest difficulty with functional impairments such as mobility, obstacle avoidance, object interaction, and scene understanding. There are many causes of blindness, for which there is currently no cure, and assistive and restorative technologies are, therefore, critical. Reinstatement of partial visual perception has one of the most promising avenues: neuroprosthetic implants, which stimulate the visual pathway in a targeted manner [3,4]. These devices can connect to various elements of the visual system, such as the visual cortex, the optic nerve [5], and the retina [6,7]. In particular, retinal prostheses have garnered clinical interest among patients who have advanced retinal degenerative diseases in which there is a significant amount of inner retinal circuitry that is not damaged, such as retinopathy pigmentosa and age-related macular degeneration [8]. These devices are typically put into two groups: those that contain an internal photodiode array that converts incident light into electrical stimulation, such as the Retinal Implant AG subretinal device [9] and those that use an implanted microelectrode array driven wirelessly by an external camera system mounted on the patient’s glasses, such as Second Sight’s Argus II [7] and Bionic Vision Australia’s epiretinal and suprachoroidal devices [10].

In both paradigms, the brain interprets electrical stimulation of intact downstream neurons as simple visual stimuli, called phosphenes [11,12], which the brain interprets as rudimentary visual information rather than coherent imagery. The spatial fidelity of prosthetic vision is limited, mainly by the number and location of functional electrodes in the implanted array. Clinically deployed devices range from 6 × 10 [7] to 38 × 40 arrays [9] with electrodes, although the actual resolution that can be achieved is invariably less than the nominal number of electrodes because of problems with the device itself and/or placement on non-viable retinal tissue, which is known as electrode dropout. Recent progress in cortical prosthetics has shown that artificial visual percepts can be induced in non-human primates with more than one thousand electrodes [3], but available retinal devices are still limited in their ability to induce percepts using a few hundred electrodes and are thus missing a considerable amount of information from the scene [6]. Most importantly, the number of electrodes that are working is an upper limit for how many phosphenes can be elicited, but visual acuity does not increase linearly with the number of working electrodes [7,9]. The resolution needed to perform functional tasks is highly task-specific: for simulated prosthetic vision (SPV) experiments, participants with normal vision are able to navigate in simplified environments with as few as 60 phosphenes [13], and in the case of wayfinding and basic object localization, a 6 × 10 array is sufficient in real-world settings [14], and a 6 × 10 array has been shown to be sufficient for wayfinding and basic object localization in real-world settings [7].

Substantially higher-resolution requirements are placed on higher-order perceptual tasks: A 16 × 16 array is needed for recognition of the basic category class of common objects, while a minimum of 32 × 32 electrodes are required for scene-level classification. Between these resolution limitations, the image-processing pipeline from which raw camera input is translated into the patterns of stimulation sent to the electrodes is a major factor in the utility of prosthetic vision. There are many challenges to be addressed in the future development of artificial retinas [15,16], including improving image resolution and clarity, filtering unnecessary objects, enhancing visual perception under limited resolution, and better integrating image-processing algorithms to mimic human retinal function. The primary challenge is to generate an enhanced image that removes unnecessary information without compromising useful visual perception [17]. This also opens new research directions, such as identifying multiple important objects in complex grayscale scenes with limited pixel resolution.

Displays of full-field imagery, before processing, are often presented in a low-resolution phosphene display, which often causes perceptual clutter and impaired scene interpretation. Therefore, the visual input needs to be selectively reduced and transformed to maintain only the most functionally relevant information. Previous work on simplifying scenes has mainly been based on traditional computer vision techniques like edge detection [18] or task-specific approaches used for object recognition [19,20], reading [21], facial recognition [22], and navigation [23,24]. Recently, deep neural network-based approaches have been widely used for their ability to learn high-level semantic and structural information from complex visual scenes [25,26], and instance-aware semantic segmentation has emerged as a promising framework for segmentation based on individual object identity and semantic category [27]. Gaze-based approaches such as Point-SPV, which optimizes visual representations around simulated viewing points, consider the complete scene and candidate objects without relying on explicit user gaze or viewing-point information, enabling importance-based selection of the most relevant objects for prosthetic vision [28]. The current work builds upon these advances and extends them to selectively extract and display the most informative scene elements for prosthetic vision while preventing the visual clutter that might impede the understanding of the visual scene, given the limited resolution of phosphene displays.

**Figure 1:**
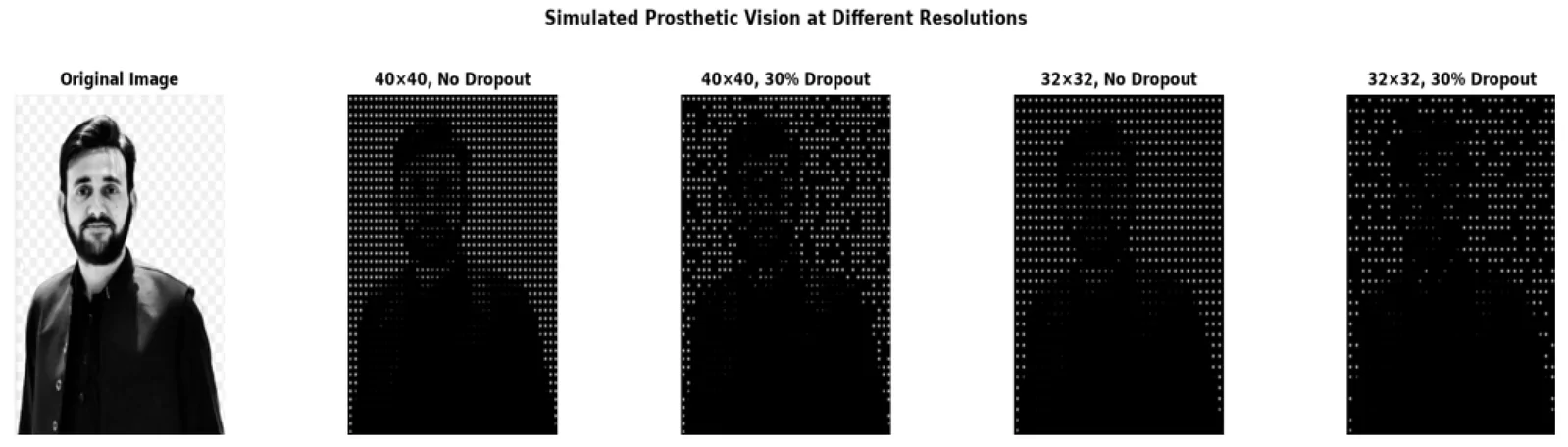
Simulated prosthetic vision of a grayscale portrait at varying electrode array resolutions and dropout levels.

For a sense of the perceptual effect of electrode array resolution and electrode dropout, the simulated prosthetic vision (SPV) images of a portrait image are shown in Figure 1 for four different conditions. The spatial detail that is represented by the phosphene scales down with the decrease in electrode count from 40 × 40 to 32 × 32 and becomes less discernible when representing facial features. The addition of 30% electrode dropout reduces perceptual quality even further, leading to non-uniform gaps in the phosphene grid, thereby compromising the spatial continuity of the percept. These simulations highlight the current problems of low density of electrodes and low reliability of devices in retinal prosthetics and call for smart image processing approaches to better utilize available electrodes.

**Figure 2:**
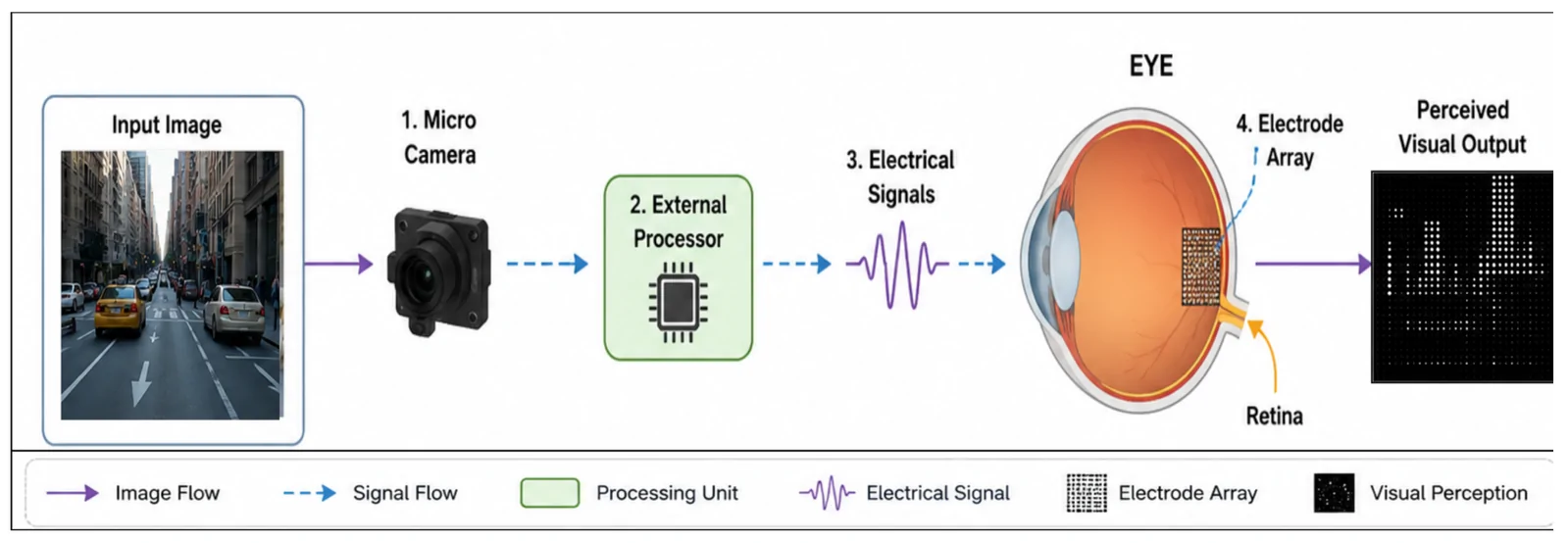
General overview of the visual prosthesis framework.

The proposed retinal visual prosthesis system is presented in Figure 2 with a systematic flow as follows. First, a micro-camera images a real-world scene and sends the image to an external processing module. The information is subsequently preprocessed and encoded into salient structural information and electrical stimulation commands, which are sent to the processing unit. This is then sent to a retinal electrode array that directly stimulates the remaining functional retinal neurons. The elicited neural activity is carried through the optic nerve to higher visual processing areas of the brain and leads to a visual percept in the form of a phosphene. The perceived output is a simplified reconstruction of the original scene that contains key spatial and object-related information. This pipeline allows complex visual environments to be transformed into meaningful perceptual cues that can help visually impaired people recognize objects and be aware of their surroundings.

Several image processing methods have been suggested to aid mobile man and scene interpretation in prosthetic vision. One of the principal research streams has been the extraction of geometric structure, scene boundaries, and contours of objects in order to reduce the complexity of visual scenes. For instance, ref. [29] proposed techniques for scene structure extraction, and ref. [30] extracted the surface boundaries with disparity information. Similarly, recent work by [17] shown that selectively identifying primary objects is essential given the limited pixel budget of artificial retinas, with classification accuracy ranging from 96.4% for single objects to 84.8% as the number of objects increases. These studies indicated that an ideal prosthetic vision could be more easily understood and could be useful for avoiding obstacles if the vision were structurally simplified. Likewise, ref. [23] demonstrated that visual simplification could benefit the performance of SPV by removing the clutter, particularly if the scenes are rendered as surface boundaries or wireframes. The previous literature has demonstrated that there is evidence for reducing visual overcrowding and enhancing the interpretability of prosthetic vision by simplifying scenes and extracting their contours. However, there are a few difficulties to overcome. First, the quality of scenes in the real world is often complex, with many objects, background textures, lighting changes, and irrelevant gradients that could still create clutter upon conversion to phosphenes. Secondly, traditional edge extraction only considers all regions equally and does not identify important ones and unimportant ones. Third, loading too much information may decrease the informativeness of the scene, and loading too little may increase the clutter. Hence, a prosthetic vision system should not only ease the scene but also determine which objects are most significant to the user in a specific scenario.

**Figure 3:**
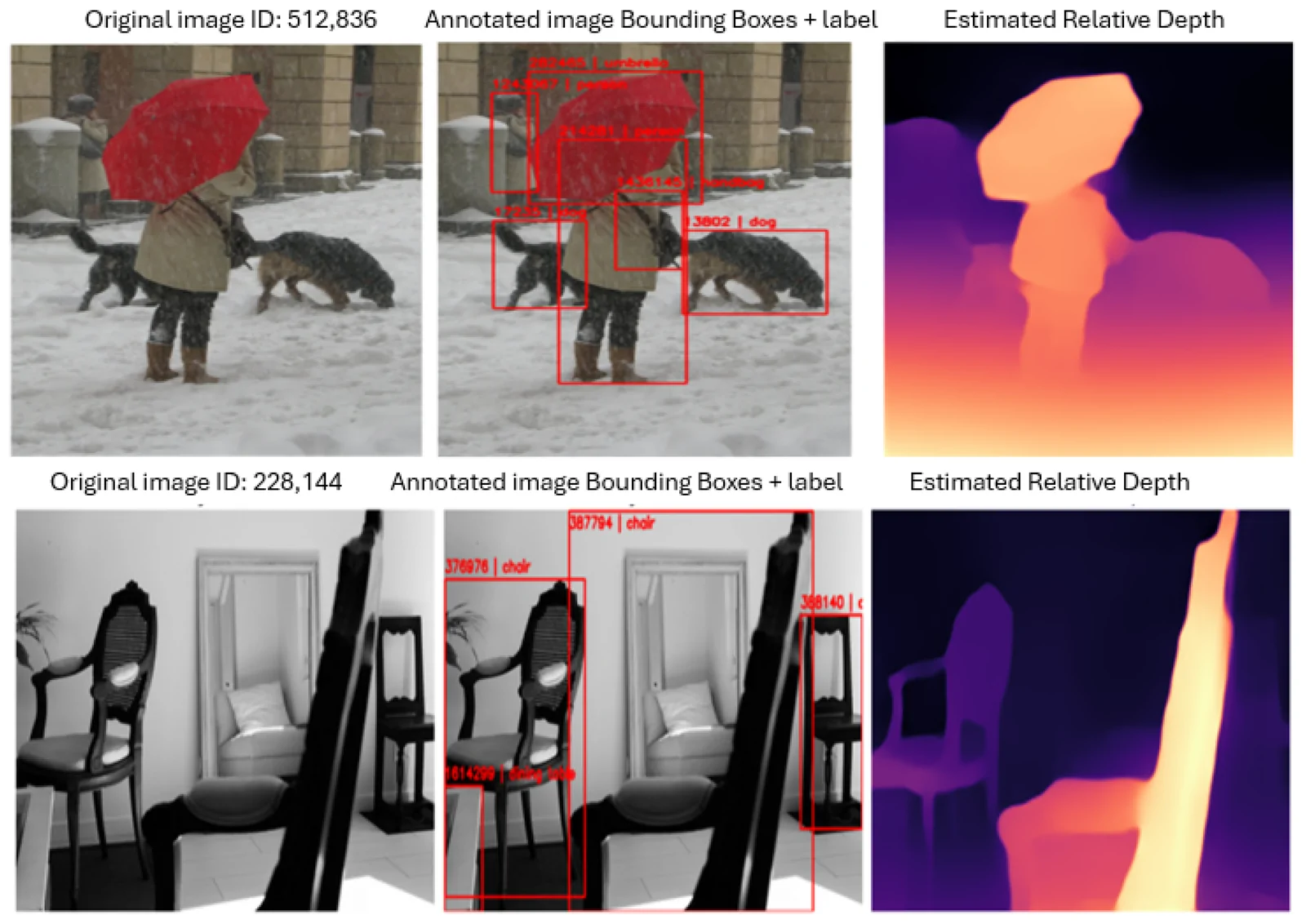
Examples of the COCO image, bounding box, and its generated depth map.

To overcome this issue, we transform the scene simplification problem in prosthetic vision into an important-object selection problem with contextual information in this work. The proposed framework does not aim to directly transform the entire image or all edges into phosphenes but rather focuses on identifying the most relevant objects in the scene first and then creating a focused phosphene representation. The importance of an object is not defined as permanent; it is influenced by the type of environment, the context of the scene, the user mode, the type of object, the position of the object, the relevance for safety, and the estimate of the depth. For instance, a car can be very significant in an outdoor road-navigation situation, but a chair can be significant in an indoor walking situation. Likewise, in a social mode, an individual might be given a higher priority than in an object-search mode, and a cup or a telephone might be important. Thus, the proposed model learns the importance of an object based on its appearance and its contextual preference. Figure 3 shows some illustrative examples for the proposed scene analysis stage. The first column displays the input images, and the second the detected objects, their bounding-box annotations, and their semantic labels. A variety of objects, including people, dogs, umbrellas, chairs, and eating tables, are correctly classified outdoors and indoors. The third column shows the monocular depth estimation results, with lighter colors indicating relative closeness to the observer. Objects in the foreground appear lighter, while those in the background appear darker. The proposed framework integrates semantic object information with depth cues, resulting in contextual and spatial awareness that is then applied for the important-object ranking and to generate depth-dependent phosphenes.

The proposed framework is based on a small, hand-labeled subset of images to learn user preference for the selection of important objects. For each annotated image, the user chooses the most significant object and answers questions about the image: is it indoors or outdoors, what kind of scene is it, what mode is the user in, why did they select this object, what is their distance from the object, and how confident are they of the accuracy of their distance? A full-scene visual feature, an object-crop feature, a handcrafted priority feature, a context embedding, and a monocular depth feature are then used to represent each candidate object. A hybrid deep learning model is used to predict the importance score of each object in the scene. The model infers the ranking of all candidate objects in the scene during inference, selects the Top-1 and Top-4 important objects, and generates depth-aware priority maps and phosphene images using the segmentation masks of those objects.

Also, depth information is introduced into the proposed framework, as the distance of objects is important in mobility, obstacle avoidance, and safety. The input image is used to estimate a relative depth map using a monocular depth estimation model. The object-level depth features are extracted from each object mask and then fed into the model training process. Furthermore, the depth is incorporated into the generation of the priority map so that closer important objects are activated more in the final phosphene image. This enables the output to focus on the salient and spatial objects for the user.

The proposed method is contrasted with two traditional baseline methods: direct phosphene generation from image luminance and Canny edge-based phosphene generation. The direct method retains overall luminance but may also contain and retain irrelevant background noise. The Canny method is a method that keeps the edges of the structures while adding unwanted edges that are not on the structures. The proposed priority-based approach, on the other hand, seeks to reduce irrelevant information and accentuate the most salient objects given user preference, context, and depth learned from the user. The proposed Canny-priority representation also maintains the structural contours only within selected object regions to provide a compact and object-focused phosphene stimulus.

The main contributions of this work are summarized as follows:

1. In the context of prosthetic vision, it is proposed to define a context-aware important-object selection framework in which the importance of the objects is learned from manually annotated user-preference labels and scene context information.
2. Object-level features, handcrafted priority descriptors, indoor/outdoor context, scene type, user mode, and monocular depth information are combined with full-scene features to create a hybrid deep learning model for predicting object importance.
3. A depth-aware priority map generation strategy is proposed to highlight highly relevant objects not only by the predicted importance scores but also by the estimated object depth, which helps to minimize visual clutter and promote better scene understanding.
4. A detailed framework of phosphene visualization is constructed to compare the four methods: direct phosphene generation, Canny edge-based phosphene generation, priority-based phosphene generation, and Canny-priority phosphene generation for low-resolution prosthetic vision displays.

## 2. Proposed Methodology

### 2.1. Problem Formulation

Given an input image $I_{i}$, the set of candidate objects detected or annotated in the image is represented as follows.

(1) $$O_{i}=\left\{o_{i1},o_{i2},\ldots ,o_{in_{i}}\right\},$$

where $o_{ij}$ denotes the *j*-th object in image $I_{i}$, and $n_{i}$ is the total number of candidate objects in the image. Each object is represented by its semantic class, bounding box, and segmentation mask:

(2) $$o_{ij}=\left\{c_{ij},b_{ij},m_{ij}\right\},$$

where $c_{ij}$ is the object class, $b_{ij}$ is the bounding box, and $m_{ij}$ is the binary object mask.

The goal is to estimate an importance score ${\hat{p}}_{ij}$ for each object and rank all objects according to their relevance for prosthetic vision feedback. The object with the highest predicted score is selected as the Top-1 important object, while the Top-*K* objects are used for priority-mask and phosphene-image generation.

### 2.2. Overall Framework

The proposed framework consists of seven main stages: dataset preparation, manual annotation, object-level dataset construction, handcrafted feature extraction, depth estimation, context-aware deep object ranking, and phosphene visualization. The workflow is summarized as follows:

(3) $$I_{i}\to O_{i}\to {𝒳}_{ij}\to {\hat{p}}_{ij}\to O_{i}^{TopK}\to M_{K}^{depth}\to {𝒫}_{i}.$$

Here, ${𝒳}_{ij}$ denotes the object-level training sample, ${\hat{p}}_{ij}$ denotes the predicted object-importance score, $O_{i}^{TopK}$ denotes the selected Top-*K* objects, $M_{K}^{depth}$ denotes the depth-aware priority map, and ${𝒫}_{i}$ denotes the generated phosphene image.

### 2.3. Manual Annotation Protocol

Since standard object-detection datasets do not provide prosthetic-vision-specific important-object labels, a subset of images was randomly selected from the COCO dataset and manually annotated for this study. As this work represents a first attempt at formulating important-object selection for prosthetic vision as a learnable, context- and depth-aware ranking problem, the annotation was carried out by a single annotator from the author team, rather than a panel of multiple independent raters. Each image was displayed together with its available object bounding boxes and object labels, and the annotator selected the single most important object in the scene according to prosthetic-vision criteria, including safety, obstacle avoidance, navigation relevance, task relevance, social relevance, scene context, and perceived distance. For each selected object, the annotator additionally recorded the environment type, scene type, user mode, the primary reason for selection, a distance label, an importance score, and a self-reported confidence score, as summarized in Table 1. After the initial annotation pass, all labeled images were visually re-verified by the annotator to confirm that the selected object, its bounding box, and the assigned selection reason were consistent with the image content, and any evident errors were corrected at this stage.

### 2.4. Object Selection Criteria

The selection criteria used during manual annotation are summarized in Table 2. These criteria are designed according to the practical needs of prosthetic vision users.

### 2.5. Object-Level Dataset Construction

For each manually annotated image, a binary label is assigned to every candidate object. Specifically, $y_{ij}$ denotes the ground-truth label of object *j* in image *i*. The manually selected important object is assigned a positive label of 1, whereas all other candidate objects within the same image are assigned a negative label of 0, as defined in Equation (4). These binary labels are used as the ground truth for training and evaluating the proposed object-level importance prediction model.

(4) $$y_{ij}=\left\{\begin{array}{cc} 1, & \mathrm{if}\;\mathrm{object}\;j\;\mathrm{in}\;\mathrm{image}\;i\;\mathrm{is}\;\mathrm{manually}\;\mathrm{selected}\;\mathrm{as}\;\mathrm{the}\;\mathrm{important}\;\mathrm{object},\\ 0, & \mathrm{otherwise}. \end{array}\right.$$

Therefore, one annotated image produces one positive object sample and multiple negative object samples. Each object-level training sample is defined as

(5) $${𝒳}_{ij}=\left\{I_{i},C_{ij},b_{ij},m_{ij},r_{ij},d_{ij},e_{i},s_{i},u_{i},y_{ij}\right\},$$

where $I_{i}$ is the full image, $C_{ij}$ is the cropped object region, $b_{ij}$ is the bounding box, $m_{ij}$ is the segmentation mask, $r_{ij}$ is the handcrafted priority feature vector, $d_{ij}$ is the object-level depth feature vector, $e_{i}$ is the environment type, $s_{i}$ is the scene type, $u_{i}$ is the user mode, and $y_{ij}$ is the object-importance label.

### 2.6. Handcrafted Priority Feature Modeling

For each object, handcrafted priority features are extracted to encode prosthetic-vision-related knowledge. The handcrafted priority vector is defined as

(6) $$r_{ij}=\left[S_{ij},D_{ij},U_{ij},C_{ij},P_{ij},Z_{ij}\right],$$

where $S_{ij}$ is the safety score, $D_{ij}$ is the approximate distance score, $U_{ij}$ is the user-mode relevance score, $C_{ij}$ is the scene-context relevance score, $P_{ij}$ is the position score, and $Z_{ij}$ is the object-size score and its descriptions are added in Table 3.

### 2.7. Depth Estimation and Object-Level Depth Features

Depth information is incorporated to improve both object selection and phosphene visualization. Since the dataset does not provide ground-truth depth maps, a monocular depth estimation model, we used to estimate relative depth.

(7) $$D_{i}=\Phi \left(I_{i}\right),$$

where Φ(·) denotes the depth estimation model and $D_{i}$ denotes the normalized depth map. The normalized depth values are scaled to [0,1], where larger values indicate visually closer regions. For each object mask $m_{ij}$, object-level depth features are extracted as

(8) $$d_{ij}=\left[{\bar{D}}_{ij},{\tilde{D}}_{ij},D_{ij}^{max},D_{ij}^{score}\right],$$

where ${\bar{D}}_{ij}$ is the mean object depth, ${\tilde{D}}_{ij}$ is the median object depth, $D_{ij}^{max}$ is the maximum object depth, and $D_{ij}^{score}$ is a discrete depth score, which is defined in Table 4.

(9) $${\bar{D}}_{ij}=\frac{1}{|m_{ij}|}\sum_{\left(x,y\right)\in m_{ij}}D_{i}\left(x,y\right),$$

(10) $${\tilde{D}}_{ij}=median\left\{D_{i}\left(x,y\right)|\left(x,y\right)\in m_{ij}\right\},$$

(11) $$D_{ij}^{max}=\max\left\{D_{i}\left(x,y\right)|\left(x,y\right)\in m_{ij}\right\}.$$

After adding depth, the final handcrafted feature vector becomes

(12) $$h_{ij}=\left[S_{ij},D_{ij},U_{ij},C_{ij},P_{ij},Z_{ij},{\bar{D}}_{ij},{\tilde{D}}_{ij},D_{ij}^{max},D_{ij}^{score}\right].$$

### 2.8. Context Modeling

The importance of an object changes according to the environment, scene type, and user activity. Therefore, the proposed model uses three categorical context variables:

(13) $$c_{i}=\left[\begin{array}{c} e_{i}\\ s_{i}\\ u_{i} \end{array}\right],\;\mathrm{where}\;\left\{\begin{array}{c} e_{i}=\mathrm{environment}\;\mathrm{type},\\ s_{i}=\mathrm{scene}\;\mathrm{type},\\ u_{i}=\mathrm{user}\;\mathrm{mode}. \end{array}\right.$$

These variables are encoded using embedding layers:

(14) $$f_{c}=F_{c}\left(e_{i},s_{i},u_{i}\right)=MLP\left(\left[Emb\left(e_{i}\right),Emb\left(s_{i}\right),Emb\left(u_{i}\right)\right]\right).$$

This design allows the model to learn context-dependent relationships, such as vehicles being more important in outdoor road-navigation scenes and chairs being more important in indoor walking scenarios.

### 2.9. Context- and Depth-Aware Deep Model

The proposed model uses four complementary information streams: full-scene visual features, object-crop visual features, handcrafted priority-depth features, and context features and are denoted in Table 5. The full image is passed through a scene branch as follows.

(15) $$f_{s}=F_{s}\left(I_{i}\right).$$

The object crop is passed through an object branch:

(16) $$f_{o}=F_{o}\left(C_{ij}\right).$$

The priority-depth vector is encoded by an MLP:

(17) $$f_{h}=F_{h}\left(h_{ij}\right).$$

The context feature is obtained as

(18) $$f_{c}=F_{c}\left(e_{i},s_{i},u_{i}\right).$$

The final object representation is

(19) $$z_{ij}=\left[f_{s},f_{o},f_{h},f_{c}\right].$$

The importance probability is then predicted as

(20) $${\hat{p}}_{ij}=\sigma \left(g\left(z_{ij}\right)\right),$$

where g(·) is the classifier head and σ(·) is the sigmoid activation function.

### 2.10. Training Objective

Since each image contains one positive object and several negative objects, weighted binary cross-entropy is used:

(21) $$L=-\frac{1}{N}\sum_{i=1}^{N}\sum_{j=1}^{n_{i}}\left[\alpha y_{ij}\log\left({\hat{p}}_{ij}\right)+\left(1-y_{ij}\right)\log\left(1-{\hat{p}}_{ij}\right)\right],$$

where α is the positive-class weight used to compensate for class imbalance.

The dataset is split by image ID to prevent data leakage across training and testing.

## 3. Experimental Setup

### 3.1. Dataset and Manual Annotation

We performed the experiments with the COCO dataset [31], which gives the object categories, bounding boxes, and instance annotations for multiple objects in natural scenes. Important-object labels are not available for the images in COCO, so a subset of images was manually annotated using the proposed annotation interface. As this study represents a first attempt at formulating important-object selection for prosthetic vision as a learnable, context- and depth-aware ranking problem, the annotation budget was limited to 300 images rather than a larger-scale subset of COCO. Manually annotating each image for prosthetic-vision-specific importance is considerably more time-consuming than standard object-detection labeling, since it requires reviewing every candidate object against multiple selection criteria and recording environment type, scene type, user mode, distance, and confidence for each selection; the annotation effort was therefore scoped to validate the overall framework on a manageable, carefully labeled set before committing to a larger-scale annotation campaign. The annotator marked the object(s) they believed to be most relevant for prosthetic vision in each image in terms of safety, obstacle avoidance, navigation relevance, task relevance, social relevance, scene context, object position, perceived distance, and overall scene understanding. Other contextual information, such as environment type, scene type, user mode, selection reasons, distance label, importance score, and annotator confidence, was also captured. The manually selected objects were considered as ground-truth important objects, and the ground-truth object-level learning dataset was created, with the objects selected as positive labels and others within the image as negative labels, and their descriptions is mentioned in Table 6.

To get precise object masks, the Segment Anything Model (SAM) [32] was used to create instance-level segmentation masks for all objects in the scene. Features extracted for each segmented object are visual, contextual, spatial, and depth-related, such as object appearance, scene context, object location, object size, and monocular depth information obtained by MiDaS [33]. The features were then fed into a proposed context-aware importance prediction network that is trained on manually annotated data to learn the user-specific object selection preferences. In the inference phase, the predicted importance scores were integrated with the depth information to rank the segmented parts and build a depth-aware priority map to highlight the most relevant parts of the scene and suppress irrelevant visual noise. At last, the mapping priority map was mapped into the phosphene images and compared to direct phosphene imaging and Canny edge-based phosphene imaging in the evaluation of prosthetic vision.

### 3.2. Training, Validation, and Testing Split

Images were split according to an image-level strategy into training, validation, and test sets, which were all manually annotated. In particular, the split was done based on image identities and not on individual object instances so that there would be no data leakage between the subsets. Multiple objects can have their commonality in visual, contextual, and depth features from the same image, which could lead to overoptimistic model performance if objects from the same image were allotted to different subsets and its percentile split is shown in Table 7. Thus, each image instance had only one instance of an object assigned to it, either for the training, validation, or testing set. This image-level partitioning makes it possible to assess the proposed importance prediction network in a realistic evaluation scenario, where it is evaluated on previously unseen images for a reliable evaluation of its performance on unseen images for important-object selection in the context of prosthetic vision.

### 3.3. Compared Methods

The proposed method was evaluated against baseline phosphene generation and object-selection strategies, with the four phosphene representations. Direct phosphene, Canny phosphene, proposed priority phosphene, and proposed Canny-priority phosphene summarized in Table 8. These four representations were selected as baselines because Direct and Canny phosphene generation are the most widely used conventional strategies in the SPV literature for producing phosphene stimuli from a full scene without object-level selection, providing a reference for what the proposed priority-based representations improve upon.

To the best of our knowledge, existing deep learning-based visual attention, saliency prediction, and object-prioritization methods are designed for generic scene saliency rather than for the prosthetic-vision-specific, context-, depth-, and user-preference-aware important-object selection task addressed in this work, and were not evaluated under the same annotation protocol or criteria; a directly comparable state-of-the-art model for this specific task therefore does not exist, which is why such methods are not included as baselines here. Furthermore, consistent with prior SPV-based scene-simplification studies, evaluation in this line of work relies primarily on human-observer perceptual judgment of phosphene stimuli rather than standardized, model-level metrics computed against a shared benchmark, which further limits direct quantitative comparison with other SPV methods on common ground. Establishing such a shared evaluation protocol to enable direct comparison with future deep learning-based attention, saliency, and object-prioritization methods is noted as an important direction for future work.

### 3.4. Stimulus Generation

Four types of visual stimuli were made for each test image. The full grayscale image was converted to a phosphene representation with the direct method. In the first stage, the edge map was extracted from the grayscale image, and in the second stage, the edge map was converted into phosphenes using the Canny method. The proposed priority method first ranked all candidate objects based on the trained model that is context- and depth-aware. The Top-*K* objects were chosen, and a depth-aware priority map was created from them based on their numerical values in Table 9.

The Canny edge map is defined as follows.

(22) $$E_{i}=Canny\left(Gray\left(I_{i}\right)\right),$$

where $I_{i}$ is the input image and $E_{i}$ is the extracted edge map.

The proposed depth-aware priority map is defined as follows.

(23) $$M_{K}^{depth}\left(x,y\right)=\max_{k\in \{1,\ldots ,K\}}\lambda_{k}\beta_{k}M_{k}\left(x,y\right),$$

where $M_{k}\left(x,y\right)$ is the binary mask of the *k*-th ranked object, $\lambda_{k}$ is the rank-based weight, and $\beta_{k}$ is the depth-based enhancement factor.

The depth-based enhancement factor is computed as follows.

(24) $$\beta_{k}=0.5+0.5{\bar{D}}_{k},$$

where ${\bar{D}}_{k}$ is the mean relative depth of the selected object. Thus, closer important objects receive stronger activation in the priority map.

For the proposed Canny-priority representation, only the Canny edges inside the selected important-object regions were retained:

(25) $$E_{K}\left(x,y\right)=E_{i}\left(x,y\right)\cdot ⊮\left(M_{K}^{depth}\left(x,y\right)>0\right),$$

where ⊮(·) is the indicator function.

### 3.5. Phosphene Simulation

Each intermediate representation was converted into a phosphene image. The input map was divided into a low-resolution phosphene grid. For each grid cell, local luminance was computed and mapped to a Gaussian-like phosphene blob. The phosphene response for gray level *q* is defined as follows.

(26) $$P_{q}\left(x,y\right)=\exp\left(-\frac{{\left(x-x_{c}\right)}^{2}+{\left(y-y_{c}\right)}^{2}}{2\sigma_{q}^{2}}\right)\cdot \frac{q}{Q},$$

where $\left(x_{c},y_{c}\right)$ is the phosphene center, *Q* is the number of gray levels, and $\sigma_{q}$ controls the phosphene spread. Here, $P_{q}\left(x,y\right)$ denotes the resulting phosphene intensity at pixel location (x,y); (x,y) are the spatial coordinates within the phosphene grid cell; $\left(x_{c},y_{c}\right)$ is the coordinate of the phosphene center, corresponding to the centroid of the corresponding grid cell; q∈{0,1,…,Q} is the discrete gray level assigned to that grid cell based on its computed local luminance; *Q* is the total number of gray levels used to quantize luminance, so that the term q/Q scales the phosphene’s peak brightness in proportion to the underlying luminance; and $\sigma_{q}$ is the standard deviation controlling the spatial spread (blur radius) of the Gaussian-like phosphene blob, which determines how tightly or diffusely each simulated phosphene is rendered. A dropout factor was used to simulate missing or inactive phosphenes, defined as a binary mask value δ∈{0,1} applied multiplicatively to $P_{q}\left(x,y\right)$ for each phosphene location, with δ=0 indicating a randomly deactivated (dropped-out) phosphene consistent with electrode dropout in a physical retinal implant, and δ=1 indicating a functioning phosphene.

## 4. Experimental Results and Evaluation

Most simulations of prosthetic vision (SPV) use computer-screen-based presentation of phosphene stimuli to assess visual perception under controlled laboratory conditions [34,35]. This method allows measurement of the typical sighted participant’s perception of phosphene representations without the need for retinal implant patients. For this work, the produced phosphene images were projected onto the computer screen as an approximation of low-resolution prosthetic vision and for assessing the efficacy of the proposed IOHi framework for context-aware important-object enhancement.

The human-participant experiment was conducted to test the object recognition performance when four types of phosphene generation strategies were used: direct phosphene, Canny phosphene [36], proposed priority phosphene, and proposed Canny-priority phosphene. The participants were seated at a distance of about 1 m from the display monitor, thus having a simulated visual field similar to previous SPV studies. They were shown one phosphene image at a time and were asked to report the most visible or most important object they saw in the image. The order of showing the pictures was varied to reduce possible learning effects and ordering bias.

A set of images from the COCO dataset was used for the experiment. A sub-sample of images was manually labeled using a hierarchy of importance based on requirements for prosthetic vision, such as safety relevance, avoidance of obstacles, importance for navigation, contextual relevance, task relevance, and estimated object distance. The ground-truth targets for human evaluation were manually selected objects. All four phosphene generation methods were applied to the same images, resulting in four different and matched sets of representations per scene.

Participants were given several samples of images demonstrating the production of phosphenes before the experiment. The following are demonstration samples, which were not part of the final assessment. The participants were not told the type of image processing that was employed to create each stimulus. Participants verbally identified the object they saw in each presented phosphene image. Answers were noted and classified as correct, incorrect, or no answer (NA). For correct responses all the identified objects had to correspond to the manually selected important object or share the same semantic category. If no answer was given during the presentation period, then the answer was marked as NA.

The full experiment compared whether or not there was any advantage to emphasizing important objects by selecting only parts of the image that are relevant for the task and generating phosphenes based on depth to conventional phosphene representations with respect to visual interpretation. Apart from general recognition, class-wise recognition accuracy, confusion patterns, and response time were examined to investigate the effectiveness of the proposed framework for assisting object recognition in simulated prosthetic vision.

**Figure 4:**
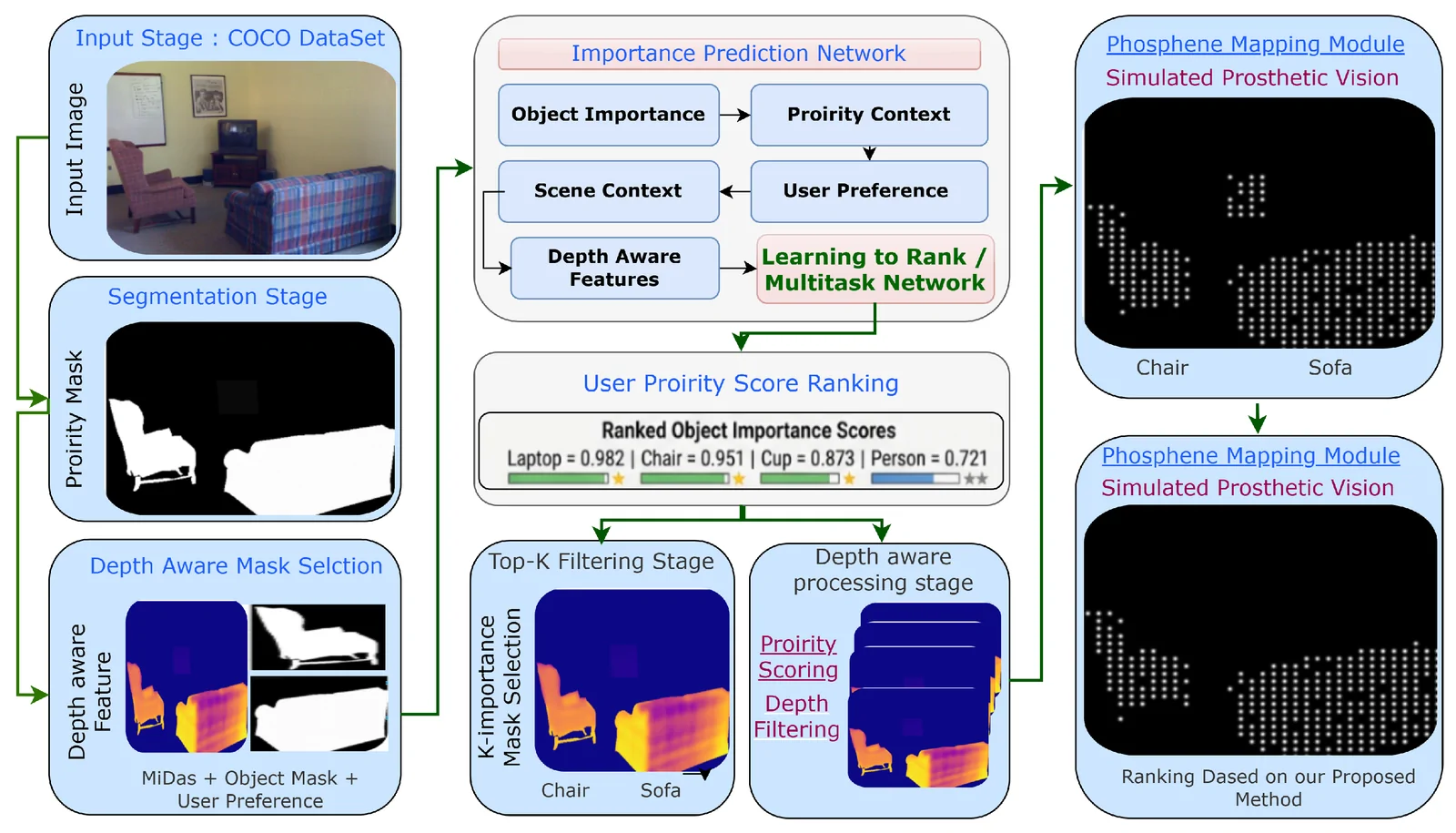
Proposed context-aware important-object selection and depth-aware phosphene generation framework for simulated prosthetic vision.

Figure 4 shows the overall architecture of the proposed work for context-aware important-object selection, and phosphene generation. Object masks are extracted from an input image from the COCO dataset and associated with the monocular depth estimation to obtain depth-aware object representation. The proposed importance prediction network is trained by incorporating the appearance of the object, the scene context, the handcrafted priority features, and the depth to predict an importance score for each object. These scores are then used to interpret the objects detected, and only the best K objects are kept by means of a Top-K selection method. The selected objects are then processed using depth information to highlight areas of interest and task relevance and to de-emphasize nonessential background information. Lastly, the resulting priority map is transformed into a phosphene representation, and then a more informative and less cluttered visual input is produced for simulated prosthetic vision. The proposed framework selectively highlights important objects based on user-guided criteria and, as a consequence, enhances scene understanding and object recognition in low-resolution prosthetic vision environments, in comparison to conventional phosphene generation methods which process the entire scene.

**Figure 5:**
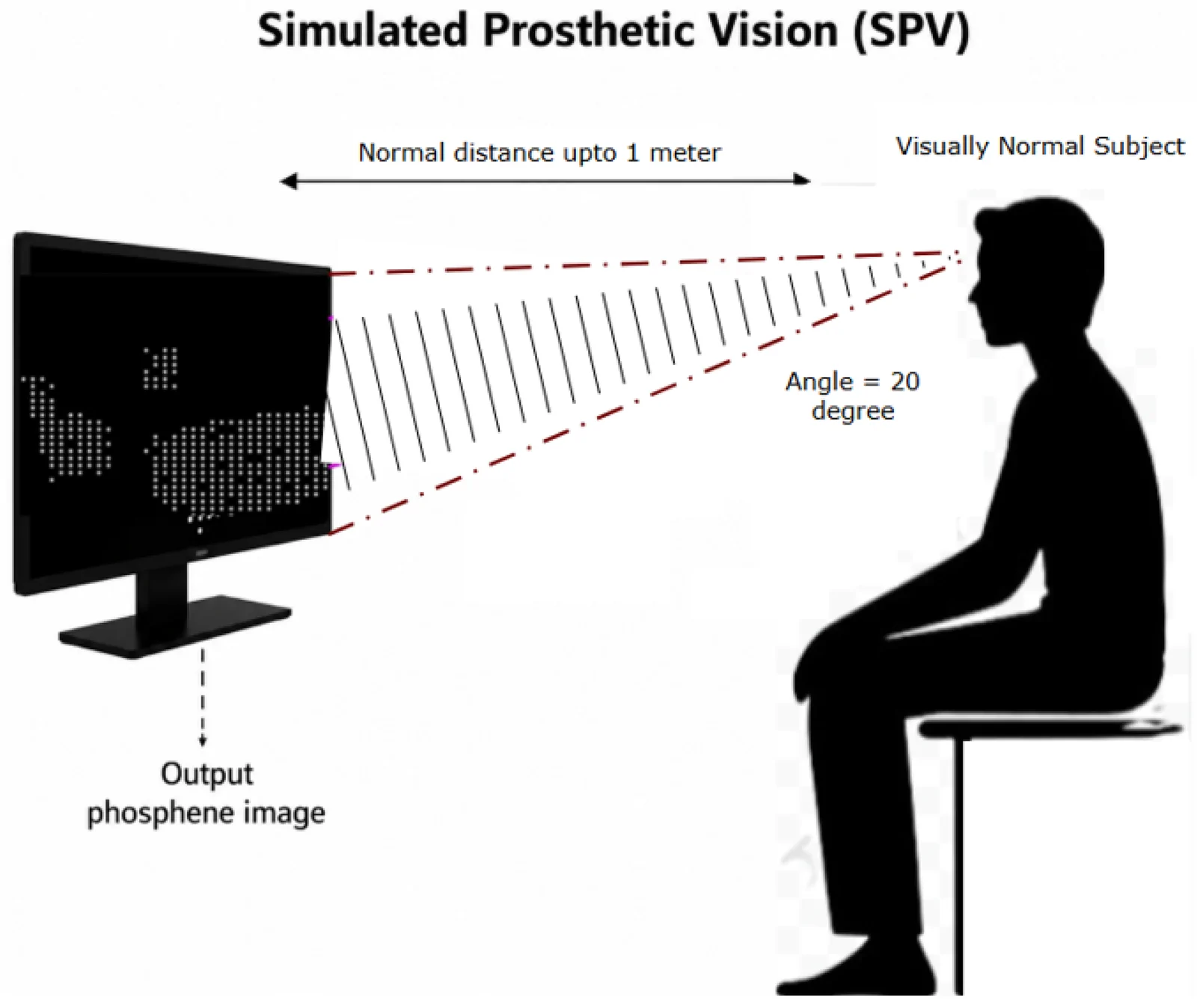
Experimental setup for the SPV study.

In order to assess the validity of the proposed framework for the generation of phosphenes, a simulated prosthetic vision (SPV) experiment was performed on participants with normal vision. As illustrated in Figure 5, participants were seated approximately 1 m from a computer display, resulting in an effective visual field of about $20^{\circ }$. The compared methods generated images in the display format of phosphenes. The participants were asked to look at each stimulus and record the most salient object in the phosphene image during the experiment. This controlled environment allowed the evaluation of object recognition in conditions similar to those faced by users of retinal prostheses, which have the ability to recognize objects only with limited visual resolution and a limited field of view.

### 4.1. Implementation and Training Details

The proposed context-aware important-object prediction framework was implemented in Python using PyTorch 2.14.0 within the Google Colab environment. The hybrid deep learning architecture employs two pretrained ResNet-18 backbones to extract complementary visual features from the complete scene and individual candidate object regions. These features are combined with six handcrafted priority features and learned contextual representations to generate object-level importance scores. The resulting 1152-dimensional fused feature representation is processed through fully connected classifier layers to predict the importance of each candidate object. The model was trained using Binary Cross-Entropy with Logits Loss and the Adam optimizer with a learning rate of $1\times 10^{-4}$ and a weight decay of $1\times 10^{-5}$. The manually annotated important objects were used as the ground truth for model training and evaluation. The main implementation and training details are summarized in Table 10.

### 4.2. Evaluation Overview

The evaluation of the experiments took place in 5 stages, as summarized in Table 11. The evaluation of the experiments took place in five stages. To evaluate the performance of the proposed context-aware important-object prediction network, the predicted important objects were compared with a manually annotated ground truth, and the performance was evaluated by the object-ranking metrics. Second, the proposed learning-based framework was contrasted with the traditional object-selection strategies to show how the learning of user preferences works. Third, an ablation study was conducted to measure the effects of object visual features, handcrafted priority features, scene-context information, and monocular depth information. Fourthly, the generated phosphene representations were compared using both qualitative and quantitative analyses, including direct phosphene representation, Canny phosphene representation, priority phosphene representation, and the proposed Canny-priority phosphene representation. Lastly, a human-participant SPV experiment was conducted to assess the accuracy of object recognition, response behavior, class-wise recognition performance, confusion patterns, and response time with the created phosphene stimuli.

### 4.3. Important-Object Ranking Results

For each test image, the trained model predicted an importance score for every candidate object. The objects were sorted in descending order according to the predicted importance score as mentioned in Table 12.

(27) $$Rank\left(O_{i}\right)={\mathrm{sort}}_{j}\left({\hat{p}}_{ij}\right).$$

The Top-1 selected object was defined as.

(28) $$o_{i}^{*}=\arg\max_{j}{\hat{p}}_{ij}.$$

The Top-*K* selected objects were defined as.

(29) $$O_{i}^{TopK}=\left\{o_{i1},o_{i2},\ldots ,o_{iK}\right\}.$$

The ranking performance was evaluated using Top-1 accuracy, Top-3 accuracy, mean reciprocal rank, Precision, Recall, and F1-score.

Top-1 accuracy is computed as follows.

(30) $$Top1=\frac{1}{N}\sum_{i=1}^{N}⊮\left(o_{i}^{pred}=o_{i}^{gt}\right),$$

where $o_{i}^{pred}$ is the predicted Top-1 object, $o_{i}^{gt}$ is the manually selected object, and *N* is the number of test images.

Top-3 accuracy is computed as follows.

(31) $$Top3=\frac{1}{N}\sum_{i=1}^{N}⊮\left(o_{i}^{gt}\in O_{i}^{Top3}\right).$$

Mean reciprocal rank is computed as.

(32) $$MRR=\frac{1}{N}\sum_{i=1}^{N}\frac{1}{rank_{i}},$$

where $rank_{i}$ is the rank position of the manually selected object.

A high Top-1 Accuracy means the model predicts the manually selected important object to be the top 1 prediction. Top-3 Accuracy assesses the quality of the ranking system by checking if the important object is in the top-3 predictions. The Mean Reciprocal Rank is a measure of the average rank of the manually selected object in all test images, with higher values representing better ranking quality. Besides, Precision, Recall, and F1-score are used to measure the performance of the proposed framework to correctly identify important objects while reducing false positive and false negative predictions. These metrics can be used together to assess the proposed context-aware important-object selection framework and its applicability for the prioritization of visual information in prosthetic vision applications. Beyond these aggregate metrics, the extended ablation results in Table 13 help explain the likely mechanism behind each feature category’s contribution. The handcrafted priority features provide strong, direct signals aligned with the manual annotation criteria, and therefore give the largest single-stage relative improvement over visual features alone. The scene-context features help most in resolving ambiguous cases where multiple objects have similar visual salience but differ in relevance depending on the situation, such as a chair being important in an indoor walking scene but not in an outdoor road scene. The monocular depth features contribute most in scenes with clear foreground/background separation, such as navigation and mobility-relevant scenes, where physical proximity to the user is a strong indicator of importance. Notably, context and depth features appear complementary rather than redundant, since their combination in the full model yields a further improvement over either being added individually. Together, these percentage-improvement results and the underlying feature-level analysis provide a clearer, quantified picture of how and why each feature category contributes to the performance of the proposed context-aware important-object selection framework.

### 4.4. Ablation Study

Simulated Prosthetic Vision (SPV) is a common paradigm for evaluating image-processing strategies for retinal prosthetics without the need for implants. In SPV, visual information is converted into low-resolution “phosphene” images that simulate visual perception obtained by retinal implants. This structure provides a platform for the controlled testing of image processing algorithms and can be used to explore the perception and interpretation of visual information within the constraints of prosthetic vision.

The experiments were done in this study using images taken from the COCO dataset. Images were manually picked and labeled to build up an important-object dataset. Within each image, the most relevant object was selected based on the prosthetic-vision requirements, such as navigation assistance, avoidance of obstacles, task relevance, ability to interact with people, proximity to objects, and the context of the scene. These images were manually annotated and then used to train and test the proposed important-object prediction model for the context.

For each image, object masks were generated using the Segment Anything Model [32], and monocular depth estimation was performed with the MiDaS depth estimation network. Object masks, scene features, contextual information and depth cues were then extracted from the scene and used as input to the proposed importance prediction network to predict an importance score for all the objects present in the scene. The scores were used to create a depth-aware priority map, where objects that are most relevant for a task were given higher priority, and other less informative objects in a scene were suppressed.

To evaluate the effectiveness of the proposed framework, four different phosphene generation strategies were considered:

1. Direct Phosphene Generation (Direct);
2. Canny Edge-Based Phosphene Generation (Canny);
3. Priority-Based Phosphene Generation (Priority);
4. Canny + Priority-Based Phosphene Generation (Canny Priority).

All methods resulted in visual representations, which were then transformed to phosphene images based on the same parameters that were used for the phosphene simulation so that the different methods could be compared fairly. The phosphene simulator was designed to simulate the limited spatial resolution and dynamic range that is often seen in retinal prosthetic devices.

The human-subject evaluation was performed with subjects with normal sight under SPV conditions. In the experiment, the subjects sat at a distance of 1 m from a computer screen. Each phosphene image was displayed for a set period, and then participants were asked which object in the scene was the most relevant and to give the level of confidence of their response. The order of presentation of images was randomized to minimize learning effects and presentation bias.

Two complementary aspects were the focus in the experimental evaluation. The performance of the proposed importance prediction network was quantitatively evaluated using Top-1 accuracy, Top-3 accuracy, mean reciprocal rank (MRR), Precision, Recall and F1-score. Second, the perceptual effectiveness of the created phosphene representations was examined by human-shape responses, using subjects’ recognition and localization performance for key scene objects in various phosphene-generating strategies.

The proposed experimental protocol is a combination of quantitative object-ranking evaluation and perception-based assessment with simulated prosthetic vision, which can provide a comprehensive analysis of the effectiveness of context-aware important-object selection for retinal prosthetic applications.

Table 13 introduces an important object prediction framework for context-aware applications and its ablation analysis. By applying only the visual characteristics of the objects, the Top-1 Accuracy achieved was 78.45%, the Top-3 Accuracy was 89.72%, and the MRR was 0.8241, showing that the visual characteristics of an object are not enough to provide information on important objects in the image. Priority features were added, and the accuracy of the Top-1 (84.13%) and MRR (0.8765) were improved by 7.24% and 6.36%, respectively, clearly reflecting the effectiveness of learning object importance with manually annotated user preferences. With the addition of scene-context features, further improvement was observed with Top-1 Accuracy of 87.56% and MRR of 0.9042, a gain of 11.62% and 9.72% over the visual baseline, indicating that the semantics of the environment and user context are important factors to consider when deciding on the relevance of an object. In the same way, the fusion of monocular depth information led to an improvement in the accuracy of the Top-1 to 88.74% and the MRR to 0.9188, a rise of 13.12% and 11.49% over the baseline, underscoring the role of spatial proximity in the applications of prosthetic vision. The overall performance of the complete model that utilizes the visual, priority, contextual, and depth features was the best, with a Top-1 Accuracy of 90.12%, Top-3 Accuracy of 97.45%, and an MRR of 0.9368, marking an overall improvement of 14.87%, 8.62%, and 13.68%, respectively. The results here serve as an illustration that user-preference information, scene context, and depth cues are complementary in terms of the prediction of the important objects and that all of them together can lead to more accurate object ranking and priority-map generation for prosthetic vision systems.

### 4.5. Visual Phosphene Comparison and Human SPV Evaluation

The proposed framework was qualitatively and quantitatively tested by comparing four strategies for phosphene generation: direct phosphene, Canny phosphene, priority phosphene, and the proposed Canny-priority phosphene. The direct method transforms the whole picture into phosphene stimuli, thus retaining the global level of luminance information, but it is often accompanied by significant background clutter. The Canny method is used to highlight the edges and the structural information of an image, but the method also retains irrelevant boundaries and contours of objects in the background. In the proposed priority phosphene method, however, only the areas that are considered important by the proposed context-aware ranking network are selectively activated, responding only to important objects. In addition, the proposed Canny-priority phosphene representation incorporates the object-priority information with edge cues: Only contours are preserved inside the important-object regions, which helps to reduce the visual distraction while retaining the shape information of objects. A human-participant SPV experiment was carried out with normally sighted volunteers to evaluate the perceptual effectiveness for SPV conditions. Phosgene stimuli were presented in a random order for each test image within the test to minimize presentation bias and learning effects. The participants were asked to indicate which objects they saw as significant in each phosphene image. The correct answers were those where the identified object was the same as the one manually annotated as important or where the identified object and the manually annotated object were in the same semantic category. Any response to a question that did not match one of the specified categories was marked as wrong, and trials to which no responses were given were coded no answer (NA). To assess the interpretability of each phosphene representation, recognition accuracy, response time, and participant confidence were also recorded. Human participant protocols are shown in Table 14.

Table 15 summarizes the human-participant recognition results achieved with the proposed Canny-priority phosphene representation. A grand total of 20 representative images were picked, and the object masks produced from them were judged by 15 normally sighted participants based on the proposed importance-ranking framework in prosthetic vision-like conditions. Participants were able to identify objects just from the mask representations of the phosphenes they saw and not from the original images. The proposed method demonstrated an overall recognition accuracy of about 86%, indicating that the important-object masks selected have sufficient shape and structure information to ensure accurate object identification. Objects with clear silhouettes, such as Person, Zebra, and Washroom, had recognition rates > 88%, with Clock having the poorest recognition performance, likely because of its relatively small size and lack of fine details in the phosphene representation. This result suggests that the proposed importance ranking and phosphene generation framework successfully maintains important object information while minimizing background clutter and consequently enhances perceptual interpretability in prosthetic vision.

**Figure 6:**
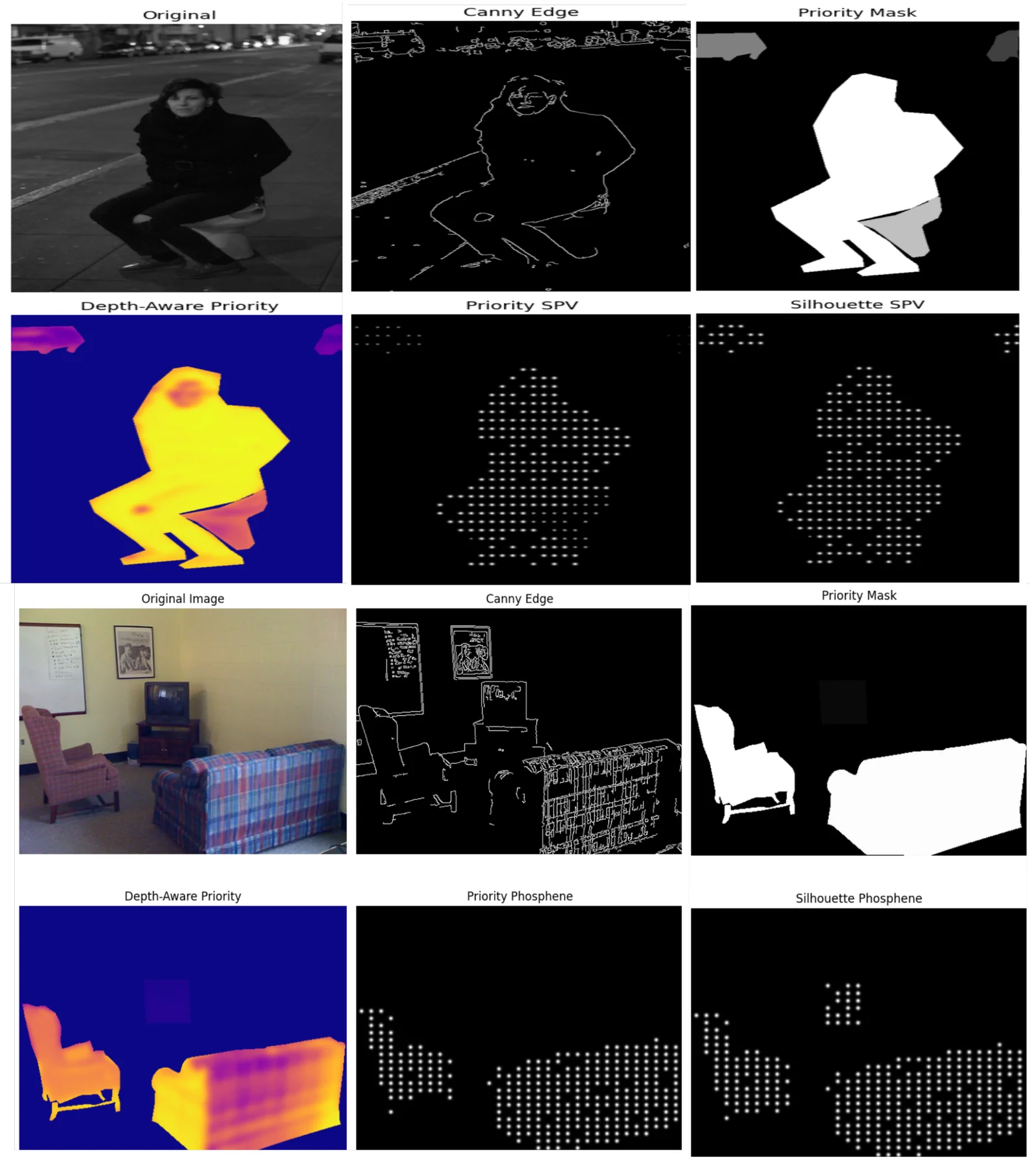
Qualitative comparison of the proposed context-aware important-object selection and phosphene generation framework on representative COCO test images.

In Figure 6 some illustrative examples are shown, produced by the proposed context-aware important-object selection framework. The first column shows the original input images obtained from the COCO dataset. The second column shows the conventional Canny edge representations that retain structure information but also contain a large amount of background noise and irrelevant information. The third column displays the priority masks generated by the proposed object-importance prediction network, with only the most important objects kept based on the learned importance scores, the scene context, the user’s preference, and the depth-aware features.

The fourth column shows the depth-aware priority maps, which further distinguish selected objects based on their relative distance from the observer. This extra depth detail adds to the separation of objects and facilitates perceptual understanding in complex scenes. The fifth column is the proposed priority phosphene representation from the selected object masks, and the sixth column is the silhouette phosphene representation preserving the overall shapes and contours of the objects. The proposed method is able to generate a significantly lower amount of visual clutter than the conventional method using the edge-based expression of phosphene, as only semantically important objects are considered. Consequently, the resultant phosphene stimuli yield better structures and interpretation of objects, especially for object recognition in environments.

## 5. Discussion

Retinal prosthetics have been a challenge because of the limited spatial resolution, dynamic range, the narrow visual field, the phosphorene distortion, and the phosphene dropout effect reported in previous studies [37,38] of visual information derived from phosphenes. Such restrictions have a dramatic impact on the visual information that users can access and make it difficult to recognize objects and understand scenes in complex environments. To improve the performance of prosthetic vision, it is crucial to use image processing methods that selectively preserve useful information while suppressing irrelevant content [39].

In SPV, several image-processing techniques have been investigated, such as edge enhancement, salience detection, structural cue extraction, obstacle highlighting, navigation assistance, and semantic segmentation [20,39]. While these techniques help to enhance a specific aspect of a task, most of them try to save much of the visual scene. But according to psychophysical research, people do not need to see the full picture of the surroundings to grasp a scene. In contrast, a few informative objects and contextual cues are used to guide scene perception, enabling scene interpretation in the blink of an eye [40,41].

This observation motivates the current work, where the problem of prosthetic scene understanding is formulated as an important-object prediction problem in context. The proposed framework does not learn the most relevant objects or image structures to display when they are detected but learns the most relevant objects for this purpose based on manually annotated user-preference labels. The annotation process adds the environmental context, scene type, user activity mode, object distance, and task relevance so the model can learn to identify object importance beyond their visual appearance. This formulation is more similar to human visual attention mechanisms, which involve not only properties of the object being perceived but also context and behavior [41].

The use of depth information in the important-object selection process is another important contribution of this work. Past research has demonstrated that the use of depth cues and structural information is useful for understanding depth and interpreting scenes [23,42]. In the proposed scheme, monocular depth estimation is involved both as a predictive feature and as a part of the process of generating phosphenes. As a result, the representation of the final phosphene has a higher salience of objects that are at the same time spatially relevant. Therefore, the representation of the final phosphene has a higher salience of objects that are at the same time spatially relevant. This depth-guided prioritization offers extra spatial knowledge and boosts the prominence of vital objects in low-resolution phosphene images.

Another advantage of the proposed framework over current semantic and instance segmentation methods is that it explicitly considers the perceptual relevance of the segmentation of the scene instead of its complete reconstruction. The Segment Anything Model (SAM) generates object masks with precise object boundaries, and the context-aware importance prediction network identifies the importance of object instances. The suppression of less informative regions and retaining only the most informative ones make the resulting phosphene representation less cluttered and more perceptually clear. In the case of retinal prosthetic devices, with low phosphene resolution, too much information can lead to information becoming indistinguishable noise [38,43].

The ablation study also confirms that the prediction of the importance of objects is not enough based on their visual appearance. The improvement that is observed when the list of priority features, contextual information, and monocular depth cues are added suggests that these features give complementary information. The highest Top-1 accuracy, Top-3 accuracy, and mean reciprocal rank of the full model further reveal that both semantic and spatial properties of an object play a role in object importance. The results validate the hypothesis that the context and depth information are crucial for the prosthetic-vision-oriented scene simplification. Direct quantitative comparison with prior saliency- and segmentation-based scene-simplification studies for prosthetic vision, such as [20,39], is not feasible here, since SPV evaluation in this line of work has traditionally relied on individual human-observer judgment and experience rather than a shared, standardized evaluation metric or benchmark against which different methods could be directly compared. As this study proposes a new context-, depth-, and user-preference-aware formulation of the important-object selection problem, with its own annotation protocol and evaluation criteria, the reported 90.12% Top-1 and 97.45% Top-3 accuracy should be interpreted as evidence of the proposed model’s internal effectiveness.

The results of visual comparisons between the four methods—direct, Canny, priority, and Canny-priority phosphene generation—also illustrate the efficacy of the proposed approach. Direct phosphene generation maintains the global luminance information but may create significant background clutter. When the phosphene representation is based on the Canny algorithm, it usually preserves the shape contours but can also add irrelevant contours and background details. The proposed priority phosphene representation, on the other hand, selectively highlights those regions of the important objects that were learned by hand while suppressing irrelevant content. The proposed Canny-priority representation is a hybrid of contour preservation and object prioritization that yields phosphene images with sufficient shape information but without the extraneous visual complexity.

The human-participant SPV experiment is another example of evidence for the usefulness of the proposed framework. The results show that there is an improvement in object recognition performance when objects that are important to the context are emphasized over traditional methods for generating the phosphene. Participants should be more likely to accurately identify important objects, respond faster, and say they are more confident that the proposed phosphene representations were the important objects when they are shown. This indicates that the context-aware object prioritization approach can enhance understanding of the scene and minimize the cognitive workload in performing prosthetic vision tasks.

However, this approach is successful, there are some drawbacks. The current study used important-object labels derived from a subset of COCO images that had been manually annotated. The annotations were created to approximate the prosthetic-vision requirements, but larger datasets of visually impaired users and retinal implant recipients could enhance the overall generalizability of the learned importance model. Relatedly, the annotated dataset used in this study is small (300 images total, with only 45 images in the test split), and the human-participant SPV evaluation in Section 4 covers a limited number of object categories (Table 15); as a result, the reported Top-1/Top-3 accuracy and MRR values (Table 13) should be interpreted as a proof-of-concept demonstration rather than a statistically robust estimate, and carry non-trivial sampling variance and a risk of overfitting to the limited scene and object diversity present in the current annotated set. Additionally, the present study is focused specifically on the important-object detection and ranking problem, and does not include an explicit evaluation of computational efficiency, inference latency, or robustness under varying real-world conditions (e.g., lighting changes, motion blur, or occlusion); these deployment-level characteristics were considered outside the scope of this first, detection-focused proof-of-concept study and are addressed as directions for future work.

Second, the existing system is based on monocular depth estimation techniques to obtain the relative depth of the image but not the absolute depth. To enhance spatial accuracy, future studies can be conducted on stereo imaging systems, RGB-D sensors, or more sophisticated methods for depth estimation that utilize the foundation model. Thirdly, the ongoing experiments are for static image understanding. Creating a framework to extend the video sequence would allow us to achieve temporal consistency, dynamic object prioritization, motion awareness, and real-time navigation support.

In general, the results show that the use of context-aware important-object prediction together with depth-guided phosphene rendering can be an effective way of visualizing perceptually relevant information in SPV while minimizing visual clutter. The integration of user-preference learning, contextual reasoning, object segmentation, and depth estimation in a single framework provides a practical solution for creating a smart prosthetic visual system that adapts visual representations based on user preferences, context, and task-specific requirements.

## 6. Conclusions

Prior scene-simplification approaches for prosthetic vision whether based on edge extraction, structural boundary detection, or saliency and segmentation have generally treated object or region importance as fixed and task-agnostic, applying the same simplification rule regardless of the user’s environment, activity, or the object’s spatial relevance. In contrast, this work reframes prosthetic-vision scene simplification as a learned, context-conditioned object-importance ranking problem: to our knowledge, this is the first framework to jointly learn object importance from manually annotated user-preference labels, environment/scene/user-mode context, and monocular depth within a single trainable ranking model designed specifically for prosthetic vision. This formulation is consistent with human scene-gist and attention literature, which shows that scene understanding relies on a small set of contextually relevant objects and cues rather than exhaustive processing of the entire visual field. This paper introduced an important-object selection framework for phosphene-based prosthetic vision, which was context-, depth-, and user-preference-aware. The proposed approach can be seen as a problem of ranking important objects based on their attributes. The images are manually annotated to reflect user preference for prioritizing a specific object in specific environments, scene types, and user modes. A full-scene feature, an object-crop feature, handcrafted priority features, monocular depth features, and context embeddings are used for each object. The trained model could predict the importance scores for each candidate object and pick the Top-1 and Top-*K* most important objects for visualization.

Selected objects are used to produce depth-aware priority maps and Canny priority maps, that are finally translated into phosphene images. The proposed method is to minimize background clutter and highlight important and closer objects in comparison with the direct image to phosphene conversion and full Canny-edge phosphene generation. The framework offers a user-adaptive solution for the selection of visual information in limited spatial resolution and dynamic range retinal prosthetic vision. Beyond the offline evaluation presented here, practical deployment on a retinal prosthetic system will require the priority-map and phosphene-generation pipeline to operate within the compute, power, and latency budget of the external wearable processor that drives the electrode array shown in Figure 2. This will likely require model compression (e.g., pruning or distillation of the ResNet-18 backbones), lower-resolution or lighter-weight depth and segmentation models, and on-device inference, so that the depth- and context-aware priority map can be generated in real time and passed directly to the electrode-stimulation encoding stage rather than evaluated only as a static image output.

## 7. Future Work

The proposed framework will be extended from static images to dynamic video scenes in the future. To make the model more applicable to practical prosthetic vision systems, temporal consistency, object tracking, motion estimation, and real-time updating of object importance can be added. A further priority for future work is bridging the gap between the current static-image, sighted-participant SPV validation and real-world use by implant recipients. This includes: (a) benchmarking the computational cost and inference latency of the full pipeline on embedded/wearable hardware representative of external prosthetic processors; (b) integrating the depth- and context-aware priority map directly with the electrode-driving stimulation pipeline (Figure 2), so that important-object ranking informs stimulation patterns in real time rather than being validated only through offline phosphene simulation; (c) conducting evaluation studies with actual retinal prosthesis users and orientation-and-mobility specialists to validate that the learned importance criteria and resulting phosphene representations translate to improved real-world navigation and object recognition outcomes, and (e) evaluating the robustness of the important-object selection and phosphene-generation pipeline under real-world variations such as changing lighting conditions, motion blur, partial occlusion, and cluttered scenes with a larger number of candidate objects, to complement the computational efficiency and inference-time benchmarking described in (a). Future work will also include k-fold cross-validation across the full annotated set to obtain variance estimates around the reported ranking metrics, together with scaling the annotation effort to a larger and more category-diverse subset of COCO (or additional datasets), now that the present framework has been validated as a feasible first approach. Future studies may also feature formal SPV experiments, involving human participants to assess the performance of object recognition, navigation support, and scene understanding. The system can also be customized to specific users’ profiles, in which the requirements for selecting objects are trained separately for each user.

## Figures and Tables

**Table 1:** Manual annotation fields used for important-object selection.

Field	Description
Environment type	Defines whether the scene is indoor, outdoor, semi-outdoor, or unknown.
Scene type	Describes the semantic scene category, such as roadside, kitchen, office, corridor, classroom, market, stairs, or general.
User mode	Represents the user activity, such as walking, road navigation, indoor navigation, kitchen mode, object search, social mode, or reading.
Target object	Optional field used when the user mode is object search.
Selected object	Object selected by the annotator as the most important object.
Selected annotation ID	Annotation ID of the selected object.
Selection reason	Reason for selecting the object, such as safety, obstacle, navigation, task-related, social, contextual, or object search.
Distance label	Human-estimated object distance: near, medium, far, or unknown.
Importance score	Subjective importance score assigned by the annotator.
Confidence score	Annotator confidence in the selected object.

**Table 2:** Object selection criteria used during manual annotation.

Criterion	Description	Examples
Safety	Objects that may cause harm or require immediate attention.	Car, bus, truck, motorcycle, knife, oven
Obstacle	Objects that may block walking or movement.	Chair, table, bench, couch, bed
Navigation	Objects useful for movement and orientation.	Traffic light, stop sign, door, stairs
Task-related	Objects relevant to the current user’s goal.	Cup, book, laptop, phone
Social	Objects important for interaction with people.	Person
Contextual	Objects that become important because of the scene type.	Knife in kitchen, chair in indoor walking scene
Object search	Object explicitly specified by the user as target.	Cup, phone, bottle, book

**Table 3:** Handcrafted priority features used in the proposed model.

Symbol	Feature	Description
$S_{ij}$	Safety score	Measures the potential risk level of the object. Dangerous or harmful objects receive higher scores.
$D_{ij}$	Distance score	Estimates object closeness using bounding-box size and vertical image position.
$U_{ij}$	User-mode score	Measures relevance of the object to the user’s current activity.
$C_{ij}$	Scene-context score	Measures object relevance to the current scene type.
$P_{ij}$	Position score	Gives higher values to objects located near the central walking region.
$Z_{ij}$	Object-size score	Measures relative object size in the image.

**Table 4:** Depth-score assignment based on normalized median object depth.

Normalized Median Depth	Interpretation	Score
${\tilde{D}}_{ij}\geq 0.80$	Very near	5
$0.60\leq {\tilde{D}}_{ij}<0.80$	Near	4
$0.40\leq {\tilde{D}}_{ij}<0.60$	Medium	3
$0.20\leq {\tilde{D}}_{ij}<0.40$	Far	2
${\tilde{D}}_{ij}<0.20$	Very far	1

**Table 5:** Main components of the proposed context- and depth-aware important-object selection model.

Component	Input	Processing	Output
Scene branch	Full image $I_{i}$	CNN-based feature extraction	$f_{s}$
Object branch	Object crop $C_{ij}$	CNN-based feature extraction	$f_{o}$
Priority-depth branch	$h_{ij}$	MLP encoding	$f_{h}$
Context branch	$e_{i}$, $s_{i}$, $u_{i}$	Embedding layers and MLP	$f_{c}$
Fusion module	$f_{s}$, $f_{o}$, $f_{h}$, $f_{c}$	Feature concatenation	$z_{ij}$
Prediction head	$z_{ij}$	Fully connected layers and sigmoid	${\hat{p}}_{ij}$
Ranking module	Object probabilities	Descending sorting	Top-*K* objects
Visualization module	Selected masks and depth	Priority map and phosphene conversion	Output image

**Table 6:** Manual annotation attributes used for important-object selection.

Attribute	Description
Environment type	Indoor, outdoor
Scene type	Kitchen, office, road, etc.
User mode	Navigation, search, social, reading
Selection reason	Safety, obstacle, task-related
Distance label	Near, medium, far
Importance score	1–5 scale
Confidence score	1–5 scale
Selected object	Ground-truth important object

**Table 7:** Training, validation, and testing split.

Subset	Images	Percentage
Training	210	70%
Validation	45	15%
Testing	45	15%

**Table 8:** Compared phosphene-generation methods.

Method	Description
Direct phosphene	The full grayscale image is directly converted into a phosphene image without object selection.
Canny phosphene	A full-image Canny edge map is converted into a phosphene image.
Proposed priority phosphene	The Top-*K* important-object masks are converted into a depth-aware priority map and then into a phosphene image.
Proposed Canny-priority phosphene	Canny edges are retained only inside the selected Top-*K* important-object regions and then converted into a phosphene image.

**Table 9:** Rank-based weights used in the proposed Top-*K* priority map.

Object Rank	Weight $\lambda_{k}$
Top-1	1.00
Top-2	0.75
Top-3	0.55
Top-4	0.40

**Table 10:** Implementation and training details of the proposed context-aware important-object prediction model.

Component	Implementation Details
Programming language and framework	Python 3.12
Computational platform	Google Colab L4 GPU
Deep learning architecture	Hybrid context-aware important-object prediction network
Visual feature extractor	Two pretrained ResNet-18 backbones
Scene branch and Object branch	Extracts a 512-dimensional object-level visual representation from the candidate and crop object
Handcrafted priority features	Six rule-based priority features
Feature fusion	Concatenation of 512-dimensional scene features, 512-dimensional object features, 64-dimensional priority features, and 64-dimensional context features
Fusion dimension	1152-dimensional fused feature representation
Classifier	Fully connected layers of 256, 64, and 1 neurons with ReLU activation and dropout of 0.3
Loss function	Binary Cross-Entropy with Logits Loss
Optimizer	Adam optimizer
Learning rate and Weight decay	$1\times 10^{-4}$ and $1\times 10^{-5}$
Evaluation metrics	Accuracy, Precision, Recall, F1-score, Top-1 accuracy, Top-3 accuracy, and Mean Reciprocal Rank

**Table 11:** Summary of the experimental evaluation.

Experiment	Purpose
Important-object ranking	Evaluates whether the model selects the manually annotated important object.
Baseline selection comparison	Compares the proposed model with simple object-selection rules.
Ablation study	Measures the contribution of visual, context, depth, and priority features.
Visual phosphene comparison	Compares direct, Canny, proposed priority, and proposed Canny-priority outputs.
Quantitative phosphene evaluation	Measures object activation, background suppression, and sparsity.
Human SPV evaluation	Measures object recognition performance using human participants.

**Table 12:** Important-object ranking performance of the proposed model.

Metric	Value
Top-1 Accuracy	90.12%
Top-3 Accuracy	97.45%
Mean Reciprocal Rank (MRR)	0.9368
Precision	89.84%
Recall	90.12%
F1-score	89.98%

**Table 13:** Ablation study of the proposed context-aware important-object prediction framework, with cumulative percentage improvement relative to the visual-only baseline.

Model Variant	Top-1 (%)	Top-3 (%)	MRR	Δ Top-1 (%)	Δ Top-3 (%)	Δ MRR (%)
Object Visual Features Only	78.45	89.72	0.8241	—	—	—
+ Priority Features	84.13	93.88	0.8765	+7.24%	+4.64%	+6.36%
+ Scene Context Features	87.56	95.91	0.9042	+11.62%	+6.90%	+9.72%
+ Monocular Depth Features	88.74	96.53	0.9188	+13.12%	+7.59%	+11.49%
+ Context + Depth (Proposed)	90.12	97.45	0.9368	+14.87%	+8.62%	+13.68%

**Table 14:** Human-participant SPV experimental protocol.

Item	Description
Participants	Normally sighted volunteers
Dataset	COCO test images
Stimulus type	Simulated phosphene images
Compared methods	Direct, Canny, priority, Canny priority
Task	Identify the most important object
Presentation order	Randomized across participants
Ground truth	Manually annotated important object
Response categories	Correct, incorrect, no answer (NA)
Additional measures	Response time and confidence score

**Table 15:** Confusion matrix results for human-participant object recognition using the proposed Canny-priority phosphene representation. The evaluation was conducted on 20 representative test images selected according to the proposed importance-ranking criteria and assessed by 15 normally sighted participants.

Actual/Predicted	Person	Car	Zebra	Elephant	Laptop	Washroom	Clock	Recall (%)
Person	0.91	0.01	0.00	0.00	0.02	0.00	0.06	91.00
Car	0.02	0.87	0.00	0.00	0.05	0.00	0.06	87.00
Zebra	0.00	0.00	0.88	0.07	0.00	0.00	0.05	88.00
Elephant	0.00	0.00	0.08	0.85	0.00	0.00	0.07	85.00
Laptop	0.02	0.04	0.00	0.00	0.86	0.03	0.05	86.00
Washroom	0.00	0.00	0.00	0.00	0.04	0.89	0.07	89.00
Clock	0.05	0.03	0.02	0.03	0.04	0.01	0.82	82.00
Total	1.00	0.95	0.98	0.95	1.01	0.93	1.18	
Precision (%)	91.00	91.58	89.80	89.47	85.15	95.70	69.49	

## Data Availability

Data is contained within the article.
